# The Role of Remote Glucose Management Using Real-time Continuous Glucose Monitoring Systems in ICU-hospitalized Patients with COVID-19

**DOI:** 10.17925/EE.2025.21.1.7

**Published:** 2025-04-28

**Authors:** Georgios Logothetis, Konstantinos Avramidis, Evanthia Konstantaki, Vasiliki Matziou, John Doupis

**Affiliations:** 1. Department of Nursing, National and Kapodistrian University of Athens, Athens, Greece; 2. Hippocratio General Hospital, Athens, Greece; 3. Department of Nursing, National and Kapodistrian University of Athens, Athens, Greece; 4. Internal Medicine and Diabetes Clinic, Iatriko Palaiou Falirou Medical Center, Athens, Greece

**Keywords:** Accuracy, clinical benefits, continuous glucose monitoring systems, COVID-19, critically ill patients, glycaemic management, intensive care units

## Abstract

The recent coronavirus disease 2019 (COVID-19) pandemic has induced many challenges in the clinical environment worldwide. In a bid to reduce the exposure of healthcare providers to severe acute respiratory syndrome coronavirus 2 and the utilization of personal protective equipment (PPE), while maintaining optimal patient care, in April 2020, the US Food and Drug Administration issued a new policy, allowing the use of continuous glucose monitoring (CGM) systems in the intensive care unit (ICU) setting. This article aimed to explore the role of real-time continuous glucose monitoring systems in patients in the ICU with COVID-19. The hybrid protocols integrating real-time CGM and point of care seem to be a feasible and safe alternative for the glycaemic management of critically ill patients with COVID-1 9, including the reduction of healthcare providers’ exposure and the preservation of PPE, whilst achieving and maintaining optimal glycaemic control.

Coronavirus disease 2019 (COVID-19) is a life-threatening infection caused by severe acute respiratory syndrome coronavirus 2 (SARS- CoV-2).^1^ Diabetes mellitus is one of the most frequent comorbidities, related to hospitalization due to SARS- CoV-2 infection, as well as a risk factor for disease severity, poor disease outcome, increased morbidity and mortality rate.^2–6^

SARS- CoV-2 enters human cells by binding to the angiotensin-converting enzyme receptor 2 (ACE2). The increase in blood glucose levels modifies the ACE2 receptor in a way that makes it more susceptible to viral binding.^7^ Hyperglycaemia itself triggers the production of inflammatory mediators (such as cytokines), which can further increase the expression or activity of ACE2 on cell surfaces, thereby making it easier for SARS- CoV-2 to attach and enter cells.^8^

Acute hyperglycaemia in patients with diabetes and COVID-19 infection may increase hospitalization duration and related morbidity, whereas effective glycaemic control in critically ill patients may positively affect the clinical outcomes of the disease.^9,10^ Furthermore, the effective management of acute hyperglycaemia in patients with COVID-19 seems to reduce viral load during the infection, although the pathogenetic mechanisms remain unknown.^11^ Adequate glucose level management plays an important role, especially in critically ill intensive care unit (ICU) patients, who receive continuous insulin infusion (CII) for hyperglycaemic crises, such as diabetic ketoacidosis (DKA) and hyperosmotic hyperglycaemic coma.^12^

CII is considered the optimal method for the management of hyperglycaemia in the ICU, as it ensures rapid onset and short action duration of the infused insulin, optimizing the glucose management of the patient in an environment with high glucose variability.^13,14^ CII procedure prerequisites accurate and hourly glucose measurements.^15^ Blood glucose assessment in hospitalized patients is usually performed by whole blood lab analysis or by point-of-care (POC) glucose measurements in arterial, venous or capillary blood samples.^16^ However, these measurements may delay the detection of abnormal glycaemic patterns and, consequently, affect the optimal insulin infusion rate.^17^ Hourly glucose measurements may also cause discomfort, blood loss and sleep disturbances.^18^ In addition, hourly POC measurements in patients with COVID-19 increase the workload and the risk of virus transmission to the healthcare personnel, also increasing the requirements for personal protective equipment (PPE).^19^ To address the new COVID-19 crisis challenges in clinical practice, the US Food and Drug Administration (FDA) has recently issued a new policy for glucose monitoring in hospitalized patients, recommending the use of non-i nvasive and remote monitoring devices, legitimizing the use of continuous glucose monitoring (CGM) in the hospital environment.^20–22^

**Table 1:** Characteristics of studies included in the systematic review^23–28^

Author, year	Study population	Type of CGM	Reference sample	Objective	Results
Chow et al., 2021^28^	30 ICU patients with COVID-19	Dexcom G6 (Dexcom, San Diego, CA, USA)	Arterial POC (Nova Statstrip^®^; Nova Biomedical, Waltham, MA, USA)	Evaluation of the accuracy and clinical benefits of rtCGM systems in the glycaemic management of patients with diabetes and severe COVID-19 infection	A 14% reduction in the average glucose sensor value during management with rtCGM, accompanied by a 50% reduction in POC measurements in 50% of patients. Additionally, 64% of nurses reported improved care for patients with diabetes, and 49% reported a reduction in the use of invasive procedures
Davis et al., 2021^23^	9 ICU patients with COVID-19	Dexcom G6	Arterial or capillary POC (Nova Statstrip)	Evaluation of a hybrid rtCGM-POC protocol integrated with an automated decision-making algorithm for CII	75.7% of the sensor values fell within 20% of the reference POC values, with a 63% reduction in POC measurements. The average TIR was calculated at 71.4%. Mechanical factors affected sensor accuracy in four patients
Sadhu et al., 2020^24^	11 ICU patients with COVID-19	Medtronic Guardian^™^ Connect (Medtronic, Northridge, CA, USA) (n=6) and Dexcom G6 (n=5)	Arterial, venous or capillary POC (Roche Accu-Chek^®^ Inform II; Roche, Basel, Switzerland)	Evaluation of the applicability and accuracy of an rtCGM system with intermittent POC checks in the glycaemic management of critically ill patients	Both rtCGM systems were easily implemented, showing acceptable accuracy (Medtronic: MARD=13.1% with 100% of values in zones A and B of the CEG; Dexcom G6: MARD=11.1% with 98% in the corresponding zones), along with good nursing acceptance
Agarwal et al., 2021^25^	11 ICU patients with COVID-19	Dexcom G6	POC	Evaluation of the accuracy and performance of rtCGM systems	MARD was calculated at 12.58%, median ARD at 6.3%, while the use of the rtCGM system resulted in approximately a 60% reduction in POC glucose measurements in patients receiving CII
Faulds et al., 2021^26^	19 ICU patients with COVID-19	Dexcom G6	Arterial or capillary POC (Nova Statstrip)	Evaluation of the safety and feasibility of a hybrid model combining rtCGM and POC glucose measurements	A 71% reduction in the number of POC measurements was observed. TIR (for glucose values between 70 and 180 mg/dL) on the first day of use was 63%, while from the second day to the seventh day, it increased to 72%. Simultaneously, there was a reduction in TBR (<70 mg/dL) from 1.5% on the first day to 0.16% on the corresponding days
Longo et al., 2022^27^	10 ICU patients with COVID-19 and 18 general ward patients^*^	Dexcom G6	POC (Roche Accu-Chek Inform II) and whole blood analysis from the laboratory (Beckman DxC 800; Beckman Coulter, Brea, CA , USA)	Evaluation of the accuracy, safety and feasibility of rtCGM systems	MARD in critically ill patients was calculated at 12.1% when the sensor glucose value was compared with both POC and laboratory values, 10.4% when compared only with the laboratory, and 12.7% when compared only with the POC

^*^In the analysis of the review, data were used only from patients in the ICU.

*CGM = continuous glucose monitoring; CII = continuous insulin infusion; COVID-19 = coronavirus disease 2019; ICU = intensive care unit; MARD = mean absolute relative difference; POC = point of care; rtCGM = real-time continuous glucose monitoring; TBR = time below range; TIR = time in range.*

Large randomized clinical trials and observational studies have already demonstrated the glycaemic benefits of CGM systems in both outpatient and hospitalized patients (*Table 1*).^23–32^ The use of a real-time CGM (rtCGM) system may provide real-time monitoring of the interstitial fluid glucose levels, transmitting the measurements approximately every 5 minutes. Furthermore, CGM systems provide alarms for out-of-range glucose values, enabling the timely detection of possible unrecognized hyper- or hypoglycaemic episodes, thereby reducing the need for frequent POC testing.^33^

In this narrative review article, we describe the clinical benefits and the accuracy of real-time continuous glucose monitoring systems in the glycaemic management of critically ill patients with COVID-19 infection, hospitalized in ICU settings.

## Accuracy

Four studies evaluated the mean absolute relative difference (MARD) of Dexcom G6, while only one study assessed the MARD of Medtronic Guardian Connect.^24–27^ MARD analysis is commonly employed to assess the accuracy of CGM systems, although it is occasionally applied to blood glucose monitoring (BGM) systems, especially when comparing the two types of systems. MARD is determined by calculating the average of the absolute relative differences between the measurement results from the CGM/BGM system and the corresponding reference method.^34^ The overall mean MARD of both rtCGM systems was reported to be 12.47%, with Dexcom G6 performing slightly better than Medtronic Guardian Connect (Mean MARD-Dexcom G6: 12.32%; MARD-Medtronic Guardian Connect: 13.1%). The MARD value was even lower for both systems for POC glucose values greater than 180 mg/dL, compared with the overall MARD for all glucose ranges.^24,26^ However, available data were limited for precisely assessing the MARD for POC measurements <70 mg/dL.^24,26,27^ Moreover, it seemed that the first day of the use of the sensor tended to provide less accurate measurements, a fact that was also observed by Faulds et al., where the overall MARD decreased from 13.9 ± 7.8% on the first day to 13.5 ± 8.1% on the second to seventh day of sensor use.^26,27^ Additionally, Faulds et al. evaluated the overall mean absolute difference, which was reported as 25 ± 16.1 and 23.2 ± 15.5% for the same time periods, respectively.^26^

In another study, MARD of Dexcom G6 was reported to be 12.58%, while the median MARD was 6.3%.^25^ Furthermore, Sadhu et al., in a study investigating both Dexcom G6 and Medtronic Guardian Connect, found that Dexcom G6 demonstrated better performance in terms of accuracy (MARD 11.1%, concordance correlation coefficient 0.89 and Pearson correlation coefficient 0.9) compared with Medtronic Guardian Connect (MARD 13.1%, concordance correlation coefficient 0.79 and Pearson correlation coefficient 0.84).^24^

The error grid (EG) analysis, a method used to evaluate the clinical accuracy of rtCGM systems, was assessed in three studies.^24,25,27^ Unlike the paired-value difference approach outlined earlier, EG analysis focuses on evaluating the clinical risk associated with the distribution of results between the investigated system and the reference method. This evaluation involves categorizing pairs of glucose measurement results into risk scores (e.g. surveillance error grid [SEG]) or risk zones (e.g. consensus and continuous glucose GDs).^34^ Clark EG analysis for both rtCGM systems showed that 98% of the paired rtCGM-POC readings were within acceptable zones A and B, while for Medtronic Guardian Connect, 100% of the readings were within zones A and B.^24,25^ In contrast, the analysis of Dexcom G6 resulted in 0.2% of pairs in zone C (readings may have the potential to result in inappropriate treatment due to the clinical impact of hyperglycaemia or hypoglycaemia), 1.2% within zone D (undetected hyperglycaemia or hypoglycaemia that requires treatment) and 0.4% within zone E (hypoglycaemia mistaken for hyperglycaemia and vice versa), while in the study of Sadhu et al. only 2% of values were within zone D.^24,25^ An SEG analysis of Dexcom G6 showed that 82.9% of rtCGM-POC matched pairs were in zone 0 (no risk), 14.1% in zone 1 (slight, lower risk), 2.7% in zone 2 (slight, higher risk) and 0.4% in zone 3 (moderate, lower risk).^27^

Only one study reported the systematic bias, which illustrates the agreement between rtCGM-POC pairs, while Sadhu et al. used the Bland–Altman plot for their analysis.^24,27^ The systematic bias was 3.5% for rtCGM-POC pairs, 0.4% for rtCGM-Lab pairs and 2.7% for rtCGM-POC-Lab pairs. The 95% limits of agreement were -30.2 to +37.2% for rtCGM-POC, -25.2 to +26% for rtCGM-Lab and -29.3 to +34.7% for rtCGM- POC- Lab pairs.^27^ In the Bland–Altman analysis, Dexcom G6 showed a mean bias of -1.94 mg/dL (p<0.001) with 95% limits of agreement between -48.74 and +44.86 mg/dL. Medtronic Guardian Connect had a mean bias of -17.76 mg/dL with 95% limits of agreement between -72.91 and +37.4 mg/dL.^24^ In the study by Longo et al., rtCGM demonstrated better accuracy measures, such as MARD, SEG analysis, mean bias and 95% agreement, when compared with Lab values (p=0.0169) rather than POC values.^27^ The overall MARD for critically ill patients was 12.7% with POC reference pairs and 10.4% with glucose reference pairs.^27^ Two studies assessed the concordance between rtCGM and POC measurements.^23,28^ Davis et al. found that for sensor glucose values above 100 mg/dL, rtCGM measurements were within 20% of the corresponding POC reference values in about 75.7% of cases. They also noted that rtCGM glucose values were lower than POC measurements during hypoperfusion states, such as shock, pulseless electrical activity, therapeutic hypothermia protocols and conditions causing sensor compression, including pronation and bathing.^23^ Similar findings were observed during compression from blood pressure cuffs.^24^ Chow et al. reported high concordance between rtCGM and arterial POC values in 93% of critically ill patients, even with significant glycaemic variability and elevated glucose levels.^28^ None of the studies reviewed reported that mechanical ventilation, steroid therapy, vasopressors, muscle relaxants, albuterol, ascorbic acid, high doses of acetaminophen, hydroxyurea or renal replacement therapy contributed to inaccuracies in CGM readings.

## Reduction of point-of-care measurements

Four studies assessed the reduction in POC measurements due to the use of rtCGM.^23–26^ On average, there was a 56.77% reduction in POC glucose testing, compared with each institution’s guideline frequency, while three studies demonstrated a reduction greater than 60%.^23,25,26^ Specifically, Sadhu et al. showed that using rtCGM systems led to a significant reduction in the frequency of POC testing. On the second day of using the rtCGM system for clinical management, the mean POC testing frequency decreased by 33.11% compared with day 0, when rtCGM values were not used. In this study, although there were POC testing frequency guidelines, there was inconsistency in nursing compliance with excessive POC testing.^24^ In another study, it was reported a reduction in POC testing in 50% of the participants, with a simultaneous increase in mean daily POC testing from 5.5 (prior to rtCGM use) to 6.9 ± 3.8 (during the use of rtCGM). This increase was attributed to the prolonged visit to the patient room for non-diabetes care, unfamiliarity and distrust of nurses in innovative monitoring technologies and the endless effort to understand COVID-19.^28^ In a study by Faulds et al., a 71% reduction in POC glucose testing was observed, with the biggest reduction occurring from the first day (mean POC testing=12) to the second day of rtCGM use (mean POC testing=7) and smaller reductions occurring daily until the fifth day (mean POC testing=4.9).^26^ Furthermore, in another study by Agarwal et al., the necessary POC testing was reduced by 60% during Dexcom G6 use, while similar findings of a 63% reduction in POC glucose testing were reported by Davis et al. when compared with the institution’s POC testing guidelines.^23,25–28,34^

Two studies evaluated the changes in mean glucose values during rtCGM for glucose management in hospitalized patients.^26,28^ Chow et al. assessed the differences in mean glucose levels measured by CGM both during (mean glucose 202.7 ± 42.1 mg/dL) and prior to the introduction of rtCGM (mean glucose 235.7 ± 42.1 mg/dL). Their results demonstrated a 14% reduction in mean glucose levels (p=0.0003) following the implementation of rtCGM, emphasizing the potential of this technology to improve glucose control in a hospital setting.^28^ In another study, Faulds et al. found that the average sensor glucose levels progressively decreased over time, with a 2.95% reduction observed when comparing glucose levels between the first day of monitoring and days 2–7.^26^ Specifically, the mean sensor glucose on the first day was 170 mg/dL, which decreased to 165 mg/dL between days 2 and 7.

## Feasibility

The feasibility of rtCGM systems was evaluated in clinical trials by examining factors such as sensor placement ease, the reliability of remote data display, app usability and external influences on data collection.

Sadhu et al. found that both Dexcom G6 and Medtronic Guardian Connect required a lengthy initiation process, including account creation on a cloud-based platform. However, sensor insertion was quick (under 5 minutes), and the display setup took 10 minutes. The Medtronic system required additional procedures and daily calibrations, while the Dexcom G6 app allowed easier input of POC glucose values.^24^ Faulds et al. reported six cases of early sensor removal or reinsertion, including accidental sensor removal (n=1), placement error (n=1), concerns about sensor accuracy (n=1) and patient discharge or death (n=3).^26^ Some complications included minimal bleeding at the insertion site.^24^ Davis et al. reported that one patient experienced bleeding at the sensor placement site, which subsequently led to sensor failure, and defibrillation during cardiac arrest caused data transmission gaps.^23^

## Reliability

The reliability of rtCGMs was evaluated by examining interruptions or gaps in the transmission of glucose data to the display device, or early sensor failure leading to sensor replacement. In a study by Sadhu et al., no data gaps or early sensor failure were reported in patients using Dexcom G6, while there was one loss of connection between the Dexcom G6 sensor and the display device and also two instances requiring sensor replacement.^24^ Furthermore, data gaps were documented during a computed tomography (CT) scan, and a sensor had to be removed for a magnetic resonance imaging (MRI) scan. In another study, after the initial placement and warm-up period, one Dexcom G6 sensor falsely displayed low measurements ranging from 40 to 55 mg/dL for 3.5 hours, resulting in early sensor replacement despite multiple calibrations.^26^

## Time in, above and below range

Three studies assessed the percentage changes in time in range (TIR), time above range (TAR) and time below range (TBR), while TBR for glucose values less than 55 mg/dL was reported in one study.^23,25,26,28^ On average, patients had a 63.1% TIR. Additionally, two other studies concluded that the TIR was at least 71.4%.^23,26^ The mean TIR (70–180 mg/dL) of Dexcom G6 was assessed in a study by Davis et al. It was reported that the TIR (70–180 mg/dL) was 71.4 ± 13.9%, the TAR for glucose values 180–250 mg/dL was 19.8 ± 9.7%, the TAR for glucose values >250 mg/dL was 7.5 ± 7.3% and the TBR (<70 mg/dL) was 0.6 ± 0.9%.^23^ In another study by Faulds et al., the TIR increased from 64 ± 23% on the first day of rtCGM use to 72 ± 16% on the second to seventh days. Concurrently, the TAR (>180 mg/dL) decreased from 34 ± 24% to 28 ± 15%, and the TBR (<70 mg/dL) decreased from 1.5 ± 4.1% to 0.16 ± 0.35%. In the same study, none of the sensor glucose values below 70 mg/dL had a corresponding POC value below 70 mg/dL, and there was only one POC measurement within the hypoglycaemic range (<54 mg/dL).^26^ Agarwal et al. found a lower TIR (46.1 ± 15.8%), higher TAR (53.3 ± 15.8%) and similar TBR (0.6 ± 0.8%) compared with other studies.^25^ In the three studies that reported percentages, the TBR was consistently ≤0.6%. In the study by Chow et al., only three patients spent 1.7–2.6% of their time below 55 mg/dL, while the other participants spent less than 1% of their time within the hypoglycaemic range for glucose values <55 mg/dL.^28^

## Nursing acceptance

In the study by Chow et al., 63% of the nurses involved in rtCGM use reported a significant improvement in the care of patients with COVID-19 and diabetes during the utilization of the devices, while 49% reduced their use of PPE.^28^ In another study, although a formal questionnaire was not used, nurses did not find rtCGM device insertion and maintenance to be a burden. Both rtCGM devices (Dexcom G6 and Medtronic Guardian Connect) were well accepted, and the ability to monitor hypo- and hyperglycaemic events was considered even better than frequent POC blood glucose testing.^24^ Additionally, integrating rtCGM systems into the ICU environment created a sense of safety when titrating insulin doses. A sense of safety was also observed in Faulds et al.’s study, where non-adjunctive insulin titrations, based on rtCGM values, increased from 41% of total insulin titrations on day 1 to 63% from the second to the fifth day of use.^26^ In many cases, there was an increased demand for more patients to use the devices. In the same study, nursing staff remained sceptical about the absolute differences between rtCGM and POC values and continued to perform POC testing according to the institution’s usual insulin infusion protocol.^24,26,27^

## Discussion

Although there are no established guidelines for an acceptable MARD value in critically ill patients, studies suggest that a CGM MARD value <14% is generally acceptable, while a MARD >18% indicates poor accuracy.^35,36^ The mean MARD reported in this systematic review (12.47%) aligns with the MARD values observed in studies that used Dexcom G6 and Medtronic Guardian Connect in non-critically ill patients (9.0–12.8%).^32,37–41^ While accuracy has not yet reached the FDA approval standards required for the non-adjunctive use of these devices in the ICU setting, the results remain promising, particularly considering the severity of illness in the patient population and the low systematic bias.

Critical illness, including factors such as mechanical ventilation, vasopressors, renal replacement therapy, hypoxia, acidosis, fever, corticosteroids and enteral and parenteral nutrition, can induce high glycaemic variability. Despite studies including patients with high Sequential Organ Failure Assessment scores, refractory hyperglycaemia and increased insulin requirements, hybrid protocols using rtCGM resulted in acceptable glycaemic control with a low incidence of hypoglycaemia, as evidenced by the time in, above and below range data.

While CGM technology has evolved over the years and overcome several limitations, it has not yet reached the point where it can completely replace POC glucose testing. rtCGM devices are still not fully capable of detecting hypo- or hyperglycaemia in certain situations (zone D), which may lead to mistreatment (zone E). Clinical factors such as hypoperfusion, sensor compression, hypothermia protocols, acetaminophen, hydroxyurea and imaging tests (e.g. MRI) can induce discrepancies between POC and rtCGM measurements. As a result, most studies have shown a reduction of up to 50% in POC-testing frequency. This reduction not only alleviates healthcare providers’ exposure to SARS- CoV- 2 but also reduces their workload.

Moreover, some of the studies included patients with medical conditions previously excluded from traditional protocols, such as those requiring mechanical ventilation, steroid therapy, vasopressors, muscle relaxants, albuterol, ascorbic acid, high doses of acetaminophen and hydroxyurea, renal replacement therapy, DKA and hyperosmolar hyperglycaemic coma. These conditions are important to be considered as they can affect glucose measurements and clinical outcomes.

Recent studies have highlighted the significant role of real-time continuous glucose monitoring in managing inpatient hyperglycaemia, particularly in critically ill patients. This technology has the potential to minimize the risks associated with traditional BGM, such as delayed results and inconsistent accuracy, while also improving patient outcomes by offering continuous data on glucose trends. As hospitals look to enhance glycaemic control in high-risk patients, the implementation of rtCGM is a promising advancement in inpatient care.^42,43^

## Conclusions

A hybrid protocol that combines rtCGM with intermittent POC measurements appears to be a feasible and safe alternative for critically ill patients. This approach helps reduce healthcare providers’ exposure to SARS- CoV- 2, preserves PPE, maintains optimal glycaemic control and enables real-time glucose monitoring. Additionally, it seems to reduce patient discomfort, blood loss and sleep interruptions. Continuous glucose monitoring can assist in identifying dangerous glycaemic changes and improve our understanding of the impact of COVID-19 on glycaemic variability. However, further studies, particularly randomized controlled trials, are needed to assess the safety, accuracy and cost-effectiveness of rtCGM in larger ICU populations. Additionally, modifications to rtCGM technology will be necessary for broader clinical use.
